# Inhibitory activity of bacterial lipopeptides against *Fusarium oxysporum* f.sp. *Strigae*

**DOI:** 10.1186/s12866-024-03386-2

**Published:** 2024-06-27

**Authors:** Mekuria Wolde Assena, Jens Pfannstiel, Frank Rasche

**Affiliations:** 1 Institute of Agricultural Sciences in the Tropics (Hans-Ruthenberg-Institute), University of Hohenheim, Garbenstr. 13, 70599 Stuttgart, Germany; 2 Department of Horticulture, Wolkite University, Wolkite, Ethiopia; 3 Core Facility Hohenheim, Mass Spectrometry Unit, University of Hohenheim, Ottilie-Zeller- Weg 2, 70599 Stuttgart, Germany; 4 International Institute of Tropical Agriculture, P.O. Box 30772-00100, Nairobi, Kenya

**Keywords:** Bacillomycin D, Lipopeptide (LP) abundance, Co-inoculation, Fos, Biological control, Microbial interaction.

## Abstract

This study investigated the influence of bacterial cyclic lipopeptides (LP; surfactins, iturins, fengycins) on microbial interactions. The objective was to investigate whether the presence of bacteria inhibits fungal growth and whether this inhibition is due to the release of bacterial metabolites, particularly LP. Selected endophytic bacterial strains with known plant-growth promoting potential were cultured in the presence of *Fusarium oxysporum* f.sp. *strigae* (Fos), which was applied as model fungal organism. The extracellular metabolome of tested bacteria, with a focus on LP, was characterized, and the inhibitory effect of bacterial LP on fungal growth was investigated. The results showed that *Bacillus velezensis* GB03 and FZB42, as well as *B. subtilis* BSn5 exhibited the strongest antagonism against Fos. *Paraburkholderia phytofirmans* PsJN, on the other hand, tended to have a slight, though non-significant growth promotion effect. Crude LP from strains GB03 and FZB42 had the strongest inhibitory effect on Fos, with a significant inhibition of spore germination and damage of the hyphal structure. Liquid chromatography tandem mass spectrometry revealed the production of several variants of iturin, fengycin, and surfactin LP families from strains GB03, FZB42, and BSn5, with varying intensity. Using plate cultures, bacillomycin D fractions were detected in higher abundance in strains GB03, FZB42, and BSn5 in the presence of Fos. Additionally, the presence of Fos in dual plate culture triggered an increase in bacillomycin D production from the *Bacillus* strains. The study demonstrated the potent antagonistic effect of certain *Bacillus* strains (i.e., GB03, FZB42, BSn5) on Fos development. Our findings emphasize the crucial role of microbial interactions in shaping the co-existence of microbial assemblages.

**Supplementary Information:**

The online version contains supplementary material available at 10.1186/s12866-024-03386-2.

## Introduction

Plants harbour a multitude of microorganisms in various ecological niches such as the rhizosphere, endosphere, and phyllosphere, creating a rich habitat for microbial diversity [1, 2]. The diverse microbial communities inhabiting these niches interact with each other and their host in complex ways, forming the plant’s endophytic microbiome [3]. The health and growth of the plant are heavily influenced by these interactions, with the host benefiting or suffering from the microbial influence [4, 5]. However, the microbiome’s functions within the plant are not solely dependent on communication with the host, but also on the intricate interactions among the microbial communities themselves. Although endophytic microorganisms co-exist within plants, our understanding of these interactions is limited. Advanced knowledge of the biochemical mechanisms underlying microbiome interactions could enable targeted exploitation of microbial functions to enhance the health and well-being of host plants.

Within plants, various fungal and bacterial species co-exist as symbionts or commensals, establishing intimate associations that can influence plant performance [6]. Endophytic fungi, in particular, provide a suitable niche for bacteria, which can utilize nutrients such as nitrogen, phosphorus, and iron, as well as organic resources such as sugar, organic acids, and amino acids released by fungal hyphae [6, 7]. On the other hand, endophytic bacteria facilitate the colonization of plants by beneficial fungi, such as arbuscular mycorrhizal fungi, and promote hyphal development by providing essential substrates such as flavonoids and furans [8–10]. For instance, *Paenibacillus validus* produces raffinose, which stimulates the growth of the endophytic fungus *Glomus intraradices* [11]. However, the co-existence of endophytic bacteria and fungi can also be antagonistic. For example, it has been reported that the endophytic fungus *Acremonium strictum* and the bacterium *Acinetobacter* sp. exhibit inhibitory interactions [12].

Endophytic microorganisms release numerous secondary metabolites that influence microbial interactions and microbiome composition within plants [13]. It is widely accepted that many microbial metabolites possess antimicrobial properties [14]. For example, non-ribosomally synthesized peptides (NRP) constitute a large group of antagonistic metabolites [15]. NRP play a crucial role in bacterial secondary metabolism [16], with cyclic lipopeptides (LP) being the most important due to their diverse biological functions [17]. *Bacillus* sp. are noteworthy examples as they play a crucial role in shaping endophytic microbiomes. They produce a diverse array of biologically active metabolites, including LP [18]. Specifically, *Bacillus* sp. are responsible for synthesizing three major families of LP. These comprise surfactins (e.g., surfactin, esperin, lichenysin, pumilacidin), iturins (e.g., iturin A, C, D and E, bacillomycin D, F and L, mycosubtilin, and bacillopeptin), and fengycins or plipastatin (e.g., fengycin A and B) [19, 20]. LP differ in their composition, length of the fatty acid moiety, and the number, type, and configuration of amino acids in the peptide portion [21]. LP have diverse biological functions, including antimicrobial properties, acting as biosurfactants, facilitating bacterial swarming and root colonization, and playing a role in biofilm formation [22, 23]. Additionally, LP are involved in the biological control of plant pathogens and enhance plant-induced systemic resistance [24, 25].

It is important to note that the co-existence of endophytic fungi and bacteria does not always result in the intended ecological outcome, such as the biological control of parasitic plants like *Striga*. One prominent example is the co-inoculation of *Fusarium oxysporum* f.sp. *strigae* (Fos), which is a putative and effective mycoherbicide for controlling *Striga* [26–28], with plant-growth promoting *Bacillus velezensis* GB03. Despite the potential for enhanced *Striga* suppression [29], co-inoculation did not yield better results compared to individual inoculation, suggesting incompatibility between the co-inoculants [30]. The incompatibility between these two potent biocontrol agents may limit their potential to be jointly applied as a microbial assemblage for controlling *Striga*.

It could be proposed that the release of bacterial LP might play a role in modulating the counteractive interplay between endophytic biocontrol agents such as Fos and *Bacillus* sp. Understanding the mechanisms underlying this dynamic interplay is crucial to enhance the efficacy of microbial assemblages for controlling parasitic organisms like *Striga*. Hence, the aim of this study is to confirm whether the presence of bacteria inhibits the growth of Fos and if the inhibition of growth is caused by the release of bacterial metabolites, specifically LP. Therefore, (i) the potential of selected endophytic bacterial strains to co-exist with Fos was evaluated, (ii) the extracellular metabolome of these bacterial strains was characterized, with a focus on LP, and (iii) the potential inhibitory effect of bacterial LP on Fos growth was investigated.

## Materials and methods

### Bacterial and fungal strains and their growth conditions

The bacterial and fungal strains used in this study and their sources are listed in Table 1. Bacterial strains were cultured on Luria-Bertani (LB) agar plates, while Fos was grown on Potato Dextrose Agar (PDA) (Carl Roth, Karlsruhe, Germany). All strains were stored at -80 °C in 40% glycerol stock solution for experimental purposes.

**Table 1:** Bacterial and fungal strains used in this work and their sources

Strains	DSM no.	Sources
*Bacillus velezensis* GB03	-	BGSC (Bacillus Genetic Stock Center), Columbus, OH, USA
*Bacillus velezensis* FZB42	23,117	Leibniz Institute DSMZ (German Collection of Microorganisms and Cell Cultures GmbH), Braunschweig, Germany
*Bacillus subtilis* BSn5	-	BGSC
*Pseudomonas protegens* CHA0	19,095	Leibniz Institute DSMZ
*Pseudomonas putida* KT2440	6125	Leibniz Institute DSMZ
*Paraburkholderia phytofirmans* PsJN	-	Austrian Institute of Technology GmbH, Tulln, Austria
*Fusarium oxysporum* f.sp. *strigae* FK3 (Fos)	-	Institute of Agricultural Sciences in the Tropics, University of Hohenheim, Germany.

### Dual-culture of Fos with bacteria

The antifungal activity of the bacterial strains against Fos was evaluated using a dual culture technique on a nutrient agar (NA) medium containing 1% peptone, 1% beef extract, 0.5% NaCl, and 15 g l^− 1^ agar. This medium was selected after optimization for the growth of both bacterial strains and Fos. For the dual culture, a 5 mm diameter disk of a 5-day-old actively growing Fos culture was placed in the center of a 90 mm NA petri dish. Bacterial cells were obtained from an overnight culture on LB agar and suspended in sterile distilled water to a concentration of 5 × 10^5^-10^6^ colony forming units (cfu) ml^− 1^. Two aliquots, each containing 5 µl of the bacterial suspensions were spot inoculated on the petri dish in opposite positions, 3 cm away from the fungal disk. Control plates were inoculated with sterile distilled water without bacterial suspension. The plates were incubated in the dark at 28 °C for 5 d. At the end of the incubation period, the diameter of the Fos culture was measured, considering the zone of inhibition between the spots of bacterial cultures. The experiment was repeated three times, with four replicates for each treatment.

### Extraction of crude lipopeptides

The crude lipopeptides (LP) were extracted using acid precipitation from Landy medium for *Bacillus* spp. and *Paraburkholderia* spp. and King’s B (KB) liquid medium for *Pseudomonas* sp [31, 32]. The Landy medium is known to promote LP production [33]. To extract the LP, an overnight culture of each bacterial strain was inoculated into 20 ml of autoclaved LB broth and incubated at 30 °C with shaking (160 rpm). Four ml of the overnight culture was transferred into a 500 ml flask containing 200 ml of Landy or KB medium and incubated at 30 °C with shaking (160 rpm) for 48 h in the dark. After incubation, the cells were removed by centrifugation (13,000 rpm) for 20 min. The supernatant was acidified to pH 2.0 using 6 M HCl and kept at 4 °C overnight to precipitate the LP. The precipitate was collected by centrifugation (13,000 rpm) for 20 min, and then extracted twice with methanol, filtered to remove any undissolved fragments, and evaporated to dryness. The crude LP was then re-dissolved in methanol to a final concentration of 10 mg ml^− 1^.

### Crude LP bioassay

The antifungal activity of crude lipopeptides (LP) extracted from bacterial strains against Fos was determined on PDA plates. The LP doses of 0, 50, 100, 150, 200, 300, and 400 µg were tested to determine Fos response (Fig. S1), and based on this assessment, 200 µg was chosen as the working concentration for the bioassay. For the bioassay, 20 µl (= 200 µg) of LP extracted from each bacterial strain was spotted on a sterile paper disc (6 mm diameter, Whatman^®^ Antibiotic Assay Discs) and allowed to dry in a laminar flow for 1 h. A 5 mm diameter agar disk of an actively growing Fos culture was placed in the centre of a PDA plate. Two LP-containing paper discs were placed on the petri dish in opposite positions at a distance of 3 cm away from the fungal disk using sterile forceps. The same amount of methanol was used for controls. The plates were incubated at 28 °C for 5 d, and the radial diameter of Fos was measured between the paper discs. Each treatment was replicated four times, and the entire experiment was repeated thrice.

The influence of crude LP on the hyphal structures of the mycelia was evaluated by bright-field microscopy. A section of hyphae was collected near the LP inoculation site and stained with lactophenol blue solution (Sigma-Aldrich, Germany). The stained samples were examined under a Leica DM750 microscope, and images were recorded using a Leica ICC50 HD camera at 40- and 60-fold magnification.

### Spore germination assay

Fos conidia were collected from PDA plates after 8 days of incubation. To obtain the conidial suspension, 10 ml of potato dextrose broth (PDB) were added to the PDA plate containing the Fos culture, and the plate was gently scraped. The suspension was then filtered through cheesecloth to remove hyphal debris. Next, 1 ml of the conidial suspension was added to 10 ml of PDB to achieve a concentration of 5 × 10^4^ spores ml^− 1^. Crude LP from each bacterial strain (100 µg ml^− 1^) were added to the suspension, and a control with no crude LP was included. The cultures were incubated in a shaker at 28 °C for 8 h. After incubation, 10 µl of the culture was placed on a hemocytometer, and at least 200 spores were counted in three separate observations using an optical microscope (Leica DM750 microscope). Spores were considered germinated if the germ tube was longer than the spore. The relative spore germination inhibition rate was calculated by comparing with the control using the following formula: germination inhibition rate (%) = [1 - (germination rate of the treatment/germination rate of the control)] × 100. This experiment was repeated thrice.

### Liquid chromatography tandem mass spectrometry (LC-MS/MS) of LP and quantification

For LC-MS/MS analysis, LP were extracted from monoculture and dual culture samples. The LP extraction was carried out with slight modifications to the method described by Kiesewalter [34]. Fos and the three *Bacillus* sp. (FZB42, BSn5, GB03) were grown in dual culture as mentioned previously. After 5 d of incubation, an 8 mm agar plug of bacterial culture was transferred to a 2 ml Eppendorf tube and extracted with 1 ml of organic solvent containing 2-propanol-ethyl acetate (1:3, v/v) and 1% formic acid. The tubes were sonicated for 1 h, and the solution was transferred to a new tube. The solvents were evaporated under N_2_, and the residue was re-dissolved in 300 µl of methanol and sonicated for 15 min. Finally, the samples were centrifuged for 3 min at 13,400 rpm, and the supernatant was transferred to an HPLC vial. The same procedure was followed for the control (monoculture), but without Fos. The extracts were stored at 4 °C until they were subjected to LC-MS/MS analysis.

Prior to LC-MS/MS analysis, methanolic extracts of crude LP from the liquid culture and LP from the inhibition zones of the three *Bacillus* sp. were gained. LC-MS/MS analysis was performed on a 1290 UHPLC system (Agilent, Waldbronn, Germany) coupled to a Q-Exactive Plus Orbitrap mass spectrometer (Thermo Fisher Scientific, Bremen, Germany) as described in Vahidinasab et al. [35]. Extracted ion chromatograms (XICs) of the corresponding LP precursor ions were generated using Compound Discoverer software version 3.3 (Thermo Fisher Scientific, San Jose, USA). Samples were analysed in triplicates. Peak areas of individual LP were calculated based on XICs of the corresponding precursor ions using Compound Discoverer 3.3 software. Assignment of individual LP was based on the precise m/z value of the precursor ion, manual inspection of corresponding MS/MS spectra and comparison with available MS/MS spectra from literature [36–39].

### Data analysis

Statistical analysis and visualization for all data were performed with the R version 4.1.1 [40]. Data were checked for normality and homogeneity of variance using Shapiro-Wilks-W-test and Levene’s test, respectively. To compare the effects of bacterial LP on mycelial growth and spore germination on Fos, as well as the dual culture data, a one-way analysis of variance (ANOVA) was performed, unless stated otherwise. Treatment means were further compared using the Tukey honesty significance difference test (HSD) using the ‘multcompView’ package. For data which did not meet the ANOVA assumption, analysis was done using Kruskal-Wallis test using ‘rstatix’ package. When applicable, multiple comparisons were done by Dunn’s test from ‘FSA’ package.

## Results

### Dual-cultivation assay of Fos with bacterial strains

**Fig. 1:**
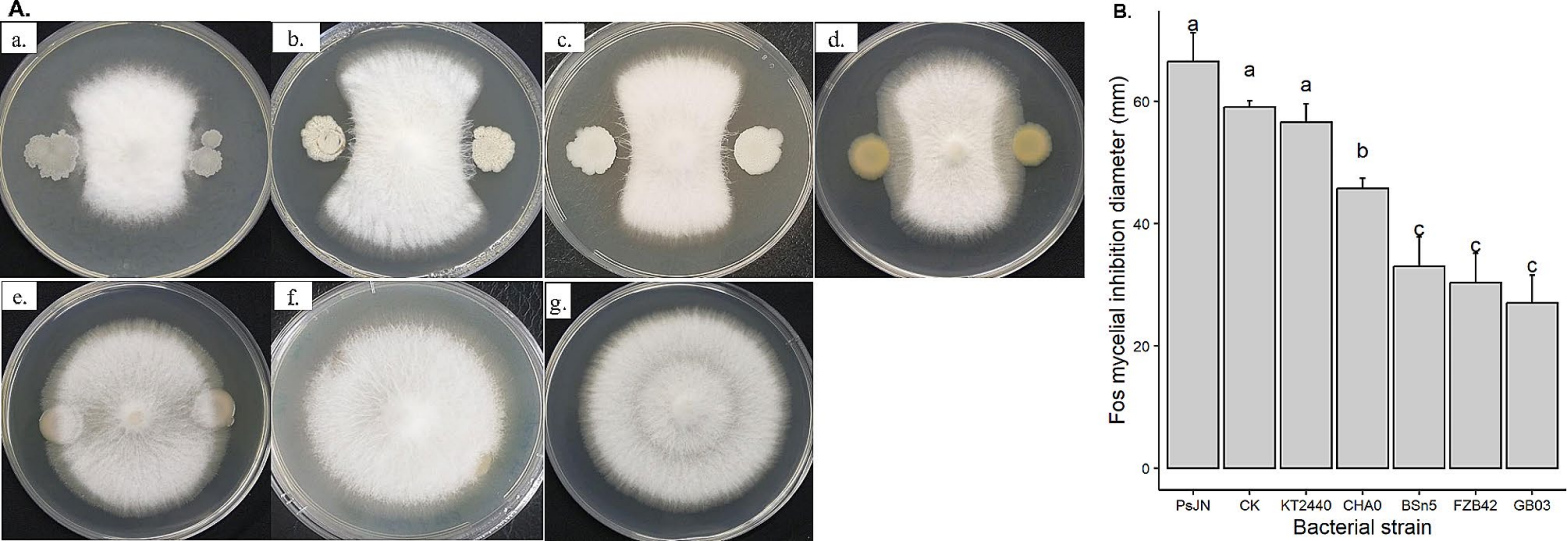
Dual culture of *Fusarium oxysporum* f.sp. *strigae* (Fos) with selected bacterial strains (*Bacillus subtilis* BSn5, *B. velezensis* GB03 and FZB42, *Pseudomonas protegens* CHA0, *P. putida* KT2440, *Paraburkholderia phytofirmans* PsJN) and the methanol control (CK) (**A**); (a) BsN5; (b) GB03; (c) FZB42; (d) CHA0; (e) KT2440; (f) PsJN; (g) CK. The inhibition diameter of Fos was presented as mean value with standard deviation, whereby columns that share a common letter do not differ significantly at the α = 0.05 level (**B**)

The results from dual culture indicate that the three *Bacillus* strains (GB03, FZB42 and BSn5) exhibited the strongest antagonism against Fos mycelial growth, as demonstrated by a clear inhibition zone (Fig. 1A-a-c). The inhibition zone revealed an average diameter of 30 mm, which was much smaller compared to the control (59 mm) (Fig. 1B) (*p* < 0.001). *Pseudomonas protegens* CHA0 exhibited an intermediate level of inhibition (46 mm inhibition zone) (*p* < 0.001) (Fig. 1A-d and B), while *P. putida* KT2440 did not show any antagonistic effect (Fig. 1A-e) as compared to the control (Fig. 1A-g). *P*. *phytofirmans* PsJN revealed the tendency to enhance Fos mycelial growth towards the bacterial culture (Fig. 1A-f and B) (*p* > 0.05).

### Antifungal activity of crude lipopeptides against Fos

**Fig. 2:**
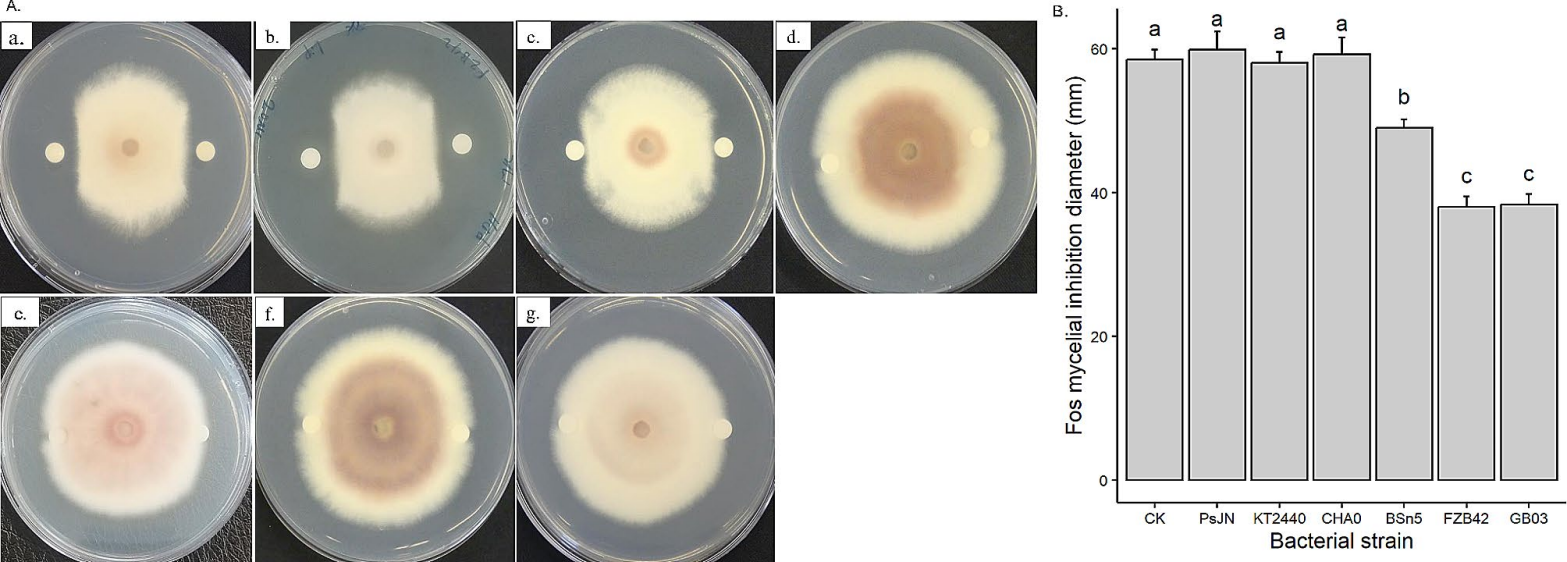
Response of mycelial growth of *Fusarium oxysporum* f.sp. *strigae* (Fos) to 20 µl of crude lipopeptides (LP) secreted by bacterial strains (*Bacillus subtilis* BSn5, *B. velezensis* GB03 and FZB42, *Pseudomonas protegens* CHA0, *P. putida* KT2440, *Paraburkholderia phytofirmans* PsJN) and methanol control (CK) (**A**); (a) GB03; (b) FZB42; (c) BsN5; (d) CHA0; (e) KT2440; (f) PsJN; (g) CK. The inhibition diameter of Fos was presented as mean value with standard deviation, whereby columns that share a common letter do not differ significantly at the α = 0.05 level (**B**)

Lipopeptides (LP) are an important class of microbial metabolites known to have antimicrobial and antifungal activity. The results from LP bioassay showed that the crude LP extracted from *Bacillus velezensis* strains GB03 and FZB42 had the largest inhibitory effect on Fos mycelial growth, with an inhibition diameter of approximately 38 mm, which was smaller than the control (58 mm) (*p* < 0.001) (Fig. 2A-a, b and B). A slight inhibition of Fos was observed with crude LP extracted from *B. subtilis* BSn5 (49 mm) (Fig. 2A-c and B) (*p* < 0.001). However, no inhibition was observed for crude LP extracted from *Pseudomonas* strains CHA0 and KT2440, *P*. *phytofirmans* PsJN, as well as the control (Fig. 2A-d-g and B).

**Fig. 3:**
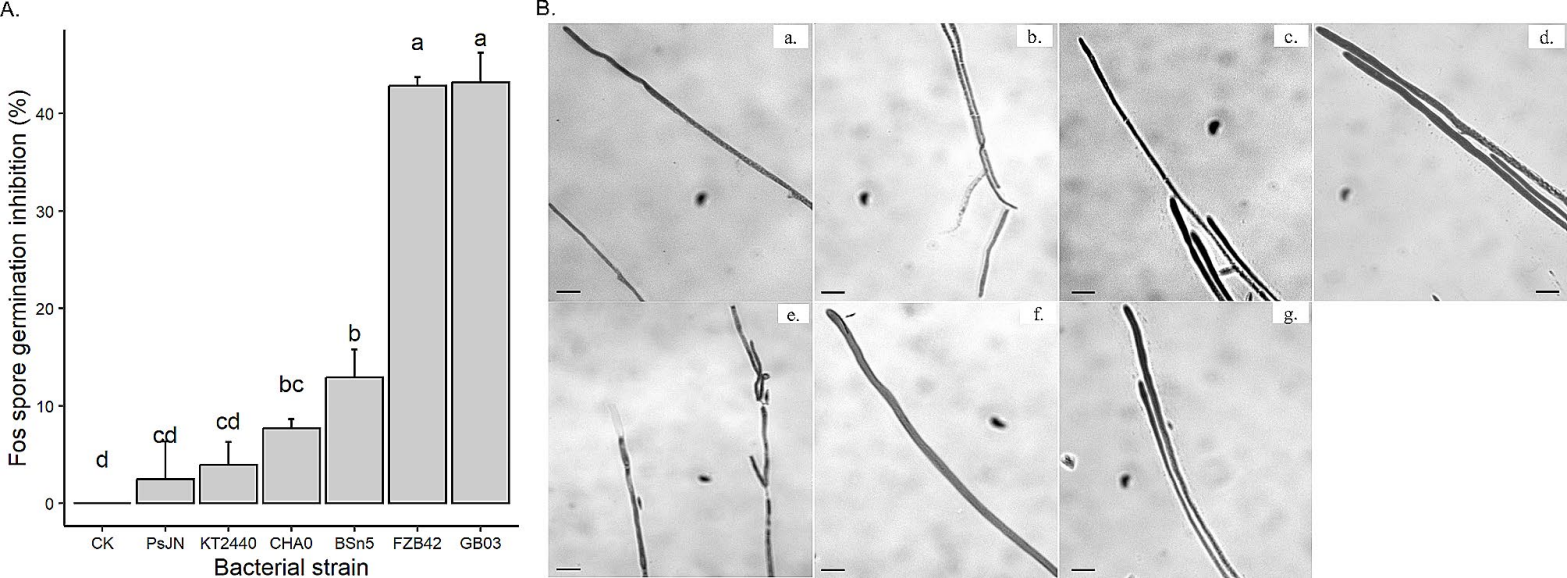
Inhibitory effect of crude lipopeptides (LP) secreted by bacterial strains (*Bacillus subtilis* BSn5, *B. velezensis* GB03 and FZB42, *Pseudomonas protegens* CHA0, *P. putida* KT2440, *Paraburkholderia phytofirmans* PsJN) and methanol control (CK) on spore germination. Data was presented as mean value with standard deviation, whereby columns that share a common letter do not differ significantly at the α = 0.05 level (**A**). Effects of crude LP on Fos hyphal structure observed with a light microscope (scale bar: 100 μm) (**B**), damaged hyphae are indicated by arrow. (a) BsN5; (b) FZB42; (c) KT2440; (d) PsJN; (e) GB03; (f) CK; (g) CHA0.

A spore germination assay was performed to evaluate the effect of crude LP on Fos development. The results showed that the spore germination rate was inhibited in the presence of crude LP (Fig. 3A). The inhibition was most pronounced for *B. velezensis* strains GB03 (43.2%) and FZB42 (42.8%), followed by *B. subtilis* BSn5 (12.9%) and *P. protegens* CHA0 (7.7%) (*p* < 0.001). However, crude LP from the other strains (PsJN, KT2440 and CHA0) did not show any significant inhibition of Fos spore germination (Fig. 3A). Furthermore, morphological analysis of Fos after exposure to crude LP extracted from *B. velezensis* strains revealed severe damage of the hyphal structure (Fig. 3B-a, b,e) as indicated by arrows. The hyphae appeared thinner, distorted, damaged and deformed. In contrast, Fos hyphae remained undamaged after treatment with methanol (control) and crude LP extracted from the other strains (Fig. 3B-c, d,f, g).

### Characterization of LP from the plate culture using LC-MS/MS

**Fig. 4:**
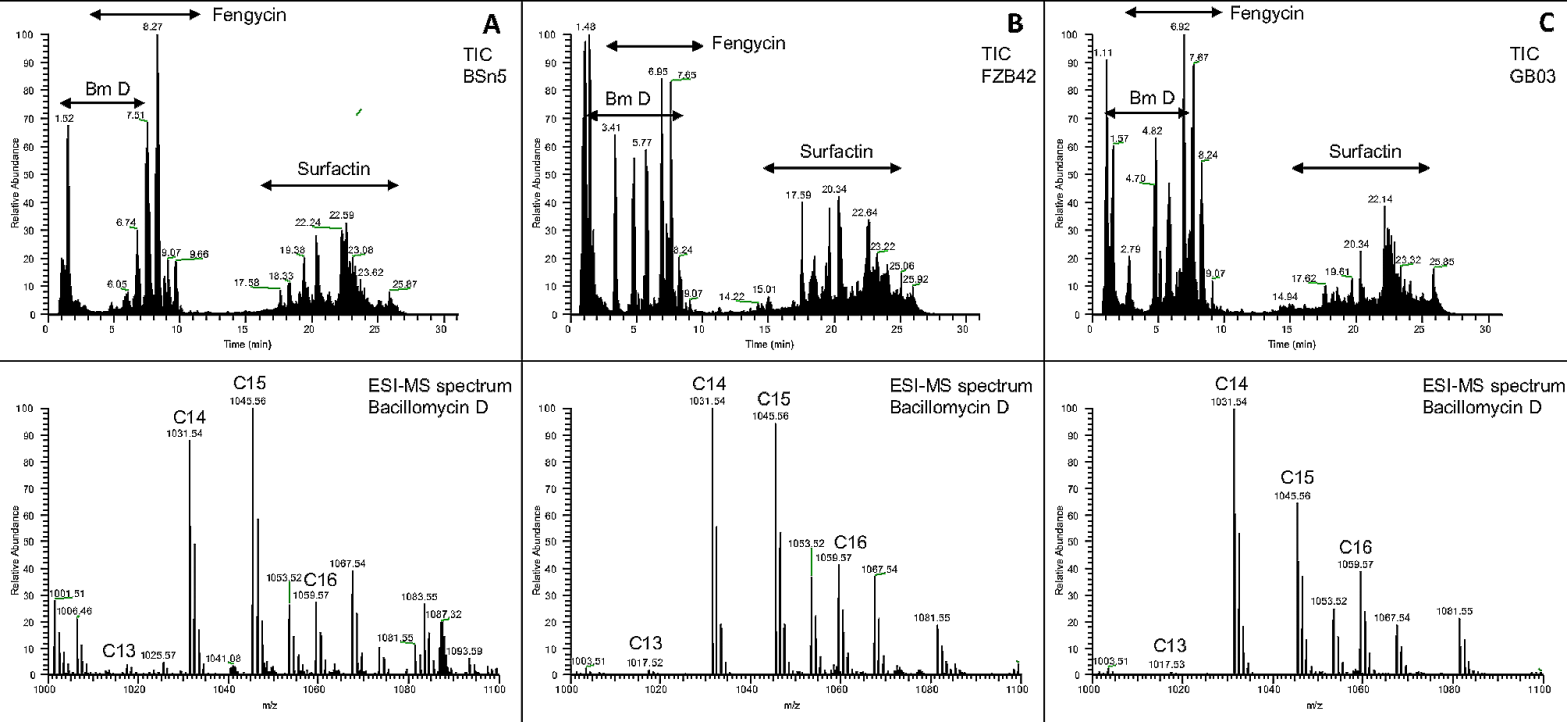
LC-ESI-MS analysis of the lipopeptide compounds produced by *B. subtilis* BSn5, *B. velezensis* FZB42 and *B. velezensis* GB03 in plate culture in the presence of *Fos*. **A**: Total ion chromatogram (TIC, upper panel) and ESI-MS spectrum (lower panel) of the extracted Bacillomycin D lipopeptides from *B. subtilis* BSn5 co-cultured with Fos. The ESI-MS sum spectrum (m/z range 1000–1100) shows Bacillomycin D lipopeptides eluted in the time interval from 1–8 min. Fatty acid chain length of different Bacillomycin D lipopeptides is indicated. **B**: Total ion chromatogram (TIC, upper panel) and ESI-MS sum spectrum (lower panel) of the extracted Bacillomycin D lipopeptides *B. velezensis* FZB42 co-cultured with Fos. The ESI-MS sum spectrum (m/z range 1000–1100) shows Bacillomycin D lipopeptides eluted in the time interval from 1–8 min. Fatty acid chain length of different Bacillomycin D lipopeptides is indicated. **C**: Total ion chromatogram (TIC, upper panel) and ESI-MS spectrum (lower panel) of the extracted Bacillomycin D lipopeptides *B. velezensis* GB03 co-cultured with Fos. The ESI-MS sum spectrum (m/z range 1000–1100) shows Bacillomycin D lipopeptides eluted in the time interval from 1–8 min. Fatty acid chain length of different Bacillomycin D lipopeptides is indicated

**Fig. 5:**
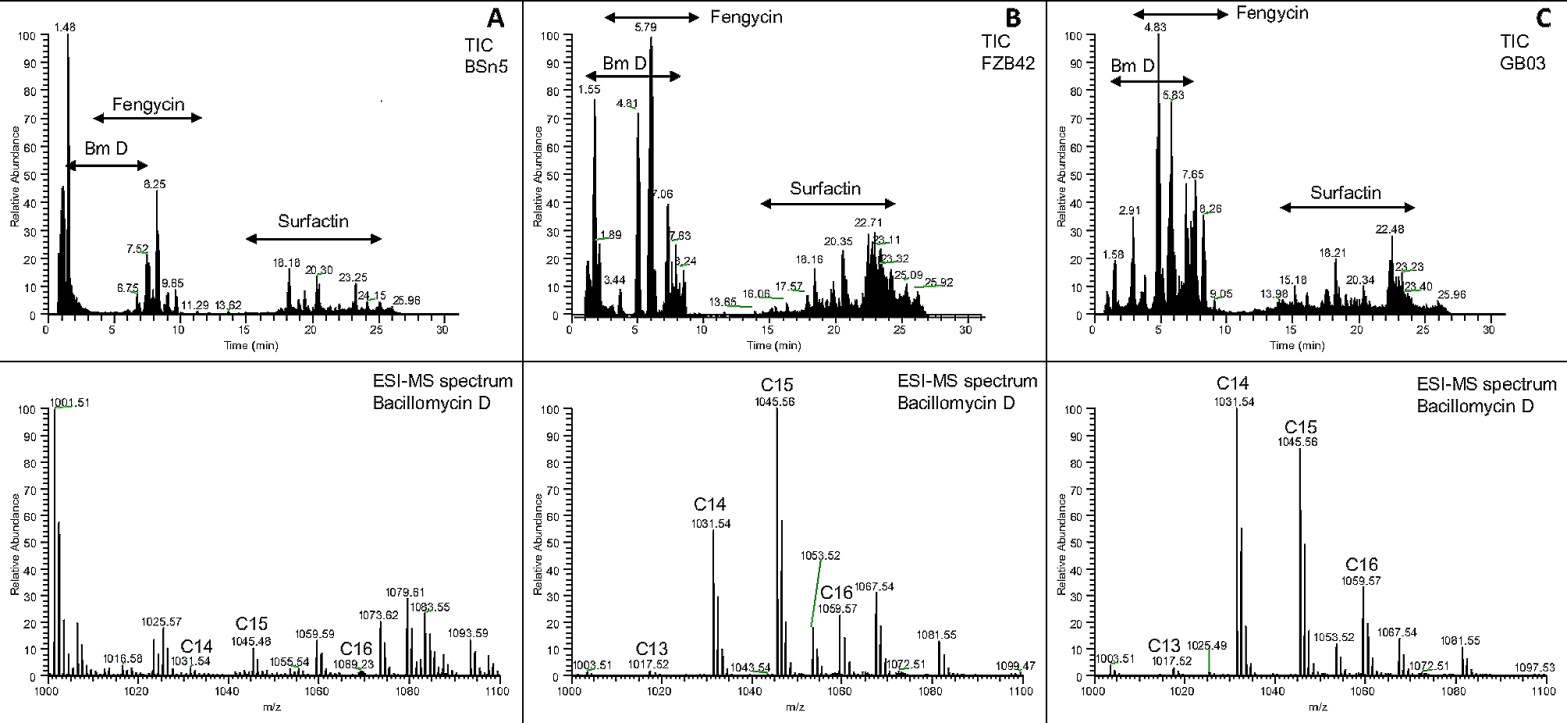
LC-ESI-MS analysis of the lipopeptide compounds produced by *B. subtilis* BSn5, *B. velezensis* FZB42 and *B. velezensis* GB03 in plate culture in the absence of *Fos*. **A**: Total ion chromatogram (TIC, upper panel) and ESI-MS spectrum (lower panel) of the extracted Bacillomycin D lipopeptides from *B. subtilis* BSn5 without Fos. The ESI-MS sum spectrum (m/z range 1000–1100) shows Bacillomycin D lipopeptides eluted in the time interval from 1–8 min. Fatty acid chain length of different Bacillomycin D lipopeptides is indicated. **B**: Total ion chromatogram (TIC, upper panel) and ESI-MS sum spectrum (lower panel) of the extracted Bacillomycin D lipopeptides *B. velezensis* FZB42 without Fos. The ESI-MS sum spectrum (m/z range 1000–1100) shows Bacillomycin D lipopeptides eluted in the time interval from 1–8 min. Fatty acid chain length of different Bacillomycin D lipopeptides is indicated. **C**: Total ion chromatogram (TIC, upper panel) and ESI-MS spectrum (lower panel) of the extracted Bacillomycin D lipopeptides *B. velezensis* GB03 without Fos. The ESI-MS sum spectrum (m/z range 1000–1100) shows Bacillomycin D lipopeptides eluted in the time interval from 1–8 min. Fatty acid chain length of different Bacillomycin D lipopeptides is indicated

To characterize the crude LP extracts in more detail and investigate the relationship between the abundance of LP and inhibitory activity, methanolic LP extracts, which have been obtained from the plate culture of FZB42, BSn5 and GB03 strains, were analysed by LC-MS/MS. In all extracts from plate culture LP of the three major LP families, namely iturin, fengycins and surfactins, were detected (Figs. 4 and 5). The result showed that bacillomycin D plays a crucial role in inhibiting Fos growth. It was detected in all strains and was the only iturin family observed, while iturin A, B or C were absent. Seven isoforms of bacillomycin D with fatty acid chain lengths from C11 to C17 were detected from the plate culture under both dual culture and monoculture conditions (Table 2). Bacillomycin D isoforms with fatty acid chain length C14 (m/z 1031.545, [M + H]^+^), C15 (m/z 1045.557, [M + H]^+^) and C16 (m/z 1059.573, [M + H]^+^) showed the highest abundance in all three bacterial strains (FZB42, BSn5, GB03) (Figs. 4 and 5). These three major isoforms comprised 94%, 89%, and 95% of all detected bacillomycin D isoforms in FZB42, GB03, and BSn5 in dual culture and 93%, 91%, and 82% in monoculture, respectively (Fig. S2). Interestingly, the total amount of bacillomycin D detected in the dual culture of FZB42 and BSn5 was much higher than their corresponding monocultures (*p* < 0.05). However, in GB03 there was no significant difference between the abundance of the major bacillomycin D isoforms under both culture conditions. The total amount of bacillomycin D isoforms was lowest in BSn5 monoculture, but a significant accumulation of bacillomycin D in the inhibition zone was recorded compared to the other LP, indicating that bacillomycin D production might be induced by the presence of Fos (*p* < 0.05).

**Table 2:** Comparison of peak area of bacillomycin D from the dual culture of the three bacterial strains with Fos and their corresponding monoculture on a PDA plate

m/z	Name	RT (min)	Cal. MW	Positive ion	Peak area (x10^5^), dual culture			Peak area (x10^5^), monoculture		
					FZB42	GB03	BSn5	FZB42	GB03	BSn5
989.495	Bacillomycin D C11	1.95	988.486	[M + H]^+^	58.2	195	66.5	25.8	221.1	17.1
1003.508	Bacillomycin D C12	2.7	1002.501	[M + H]^+^	208.1	991.6	165.7	219.9	1863.9	1
1017.526	Bacillomycin D C13	3.5	1016.518	[M + H]^+^	148	598.51	62.7	77.7	1199.4	0.4
1031.545	Bacillomycin D C14	4.95	1030.533	[M + H]^+^	9286.7	24083.4	4089.4	92.1	31946.5	55.3
1045.557	Bacillomycin D C15	5.9	1044.549	[M + H]^+^	13685.8	25538.3	13,528	9102.3	36639.8	39.3
1059.573	Bacillomycin D C16^+^	7.2	1058.566	[M + H]^+^	1985.4	7876.1	2454	1116.5	7807.5	6
1059.573	Bacillomycin D C16	7.45	1058.566	[M + H]^+^	2130.8	9761.6	925.9	1046.2	10135.2	5.4
1073.588	Bacillomycin D C17	8.35	1072.582	[M + H]^+^	1158.2	6307.3	696.2	492.3	4427.9	4.37
Total bacillomycin D					28660.9ab	75351.9a	21988.4ab	12172.5b	94241.4a	128.9bc

+ represents isomers which showed two peaks. Means followed by the same letter are not significantly different (Kruskal-Wallis test with Dunn’s multiple comparison test, *P* < 0.05)

For all bacterial strains, several surfactin and fengycin isoforms were detected in monoculture and dual culture with Fos. However, no significant differences in abundance of the two isoforms were observed between monoculture and dual culture. This suggested that their production was not influenced by the presence of Fos. Surfactin isoforms were detected within m/z range from m/z 994.642 to m/z 1064.722 [M + H]^+^, corresponding to surfactin variants with fatty acid chains length from C12 to C17, and were produced in high quantities by all strains under both culture conditions. The most abundant surfactin variants in all strains and culture conditions were surfactin C15 (m/z 1036.691, [M + H]^+^) and surfactin C14 (m/z 1022.675, [M + H]^+^).

Fengycin isoforms A1, A2, B1 and B2 [38] were likewise observed in all strains and under both culture conditions (Table 3). Fengycin isoforms A1 and B1 were detected with saturated and unsaturated fatty acid chains, while isoforms A2 and B2 were only identified with saturated fatty acid chains. Fengycin isoforms with saturated and unsaturated fatty acid chains were detected in the m/z range of m/z 1435.771 to m/z 1519.868 and m/z 1447.826 to m/z 1489.856, respectively, with the saturated fatty acid variants being more abundant (Table 3). In addition to the protonated molecular ions [M + H]^+^, also doubly charged [M + 2 H]^2+^ fengycin molecular ions were detected in samples from the plate culture, which has also been observed in other studies [41, 42].

**Table 3:** Comparison of peak area of Fengycin from the dual culture of the three bacterial strains with Fos and their corresponding monoculture on a PDA plate

m/z	Name	RT (min)	Cal. MW	Positive ion	Peak area (x10^5^), dual culture			Peak area (x10^5^), monoculture		
					FZB42	GB03	BSn5	FZB42	GB03	BSn5
1435.771	Fengycin A1 C14 sFA	5.2	1434.765	[M + H]^+^	84	916.1	66	93.1	795.9	11.1
1449.785	Fengycin A1 C15 sFA	6	1448.779	[M + H]^+^	141.2	888.8	189.9	46.5	741.9	40
725.397	Fengycin A1 C15 sFA	5.8	1448.779	[M + 2 H]^2+^	832.3	977.8	335	758.7	2613.3	122.7
1447.809	Fengycin A1 C15 usFA	8.45	1446.801	[M + H]^+^	177.5	391.3	181.9	160.6	320.3	223.1
1463.803	Fengycin A1 C16 sFA	7	1462.795	[M + H]^+^	1635.1	5658.6	967	792.4	6960.7	331
1461.826	Fengycin A1 C16 usFA	9.2	1460.819	[M + H]^+^	18.9	59.5	155.7	67.5	74	86.2
1477.818	Fengycin A1 C17 sFA	7.6	1476.812	[M + H]^+^	276.6	2099.4	248.5	1279.2	3595.8	1681.6
739.413	Fengycin A1 C17 sFA	7.5	1476.812	[M + 2 H]^2+^	3963.3	15,590	6803.3	1573.3	17333.3	2770
1449.788	Fengycin A2 C16 sFA	6.45	1448.781	[M + H]^+^	91.1	629.8	53.2	69.5	813.9	7.2
1463.803	Fengycin A2 C17 sFA	7.2	1462.797	[M + H]^+^	42.1	366.6	474.6	24.7	172.9	30.3
1491.837	Fengycin A1 C18 sFA	8.8	1490.829	[M + H]^+^	167.3	259.3	54.6	62	231.3	41.9
1435.772	Fengycin A2 C15 sFA	5.3	1434.764	[M + H]^+^	21.8	339.3	22.6	32.9	224.7	1.1
1463.801	Fengycin B1 C14 sFA	5.95	1462.794	[M + H]^+^	36.1	536.4	41.5	40.8	468.8	13.4
1461.826	Fengycin B1 C14 usFA	8.25	1460.818	[M + H]^+^	1.6	12.2	14.8	1.6	11.7	5.9
1477.819	Fengycin B1 C15 sFA	6.65	1476.812	[M + H]^+^	54.7	705.3	84.4	85.6	625	55.9
1475.842	Fengycin B1 C15 usFA	9.05	1474.835	[M + H]^+^	64.4	168.6	189.5	81.9	126.4	125.4
1491.834	Fengycin B1 C16 sFA^+^	7.55	1490.828	[M + H]^+^	102.4	2459.5	879.6	196.2	1640.9	524.3
1489.856	Fengycin B1 C16 usFA	9.7	1488.850	[M + H]^+^	8.1	18	8.3	44.9	6.4	51.5
1491.834	Fengycin B1 C16 sFA	7.6	1490.828	[M + H]^+^	1156	7420	330.3	434.4	6393.3	660.3
1505.85	Fengycin B1 C17 sFA	8.45	1504.843	[M + H]^+^	229	3746.4	2525.2	1086.3	2488.6	1910.8
1463.803	Fengycin B2 C15 sFA	6.9	1462.797	[M + H]^+^	55.6	293.9	104.4	251	357.4	254.9
1519.868	Fengycin B1 C18 sFA^+^	9.2	1518.861	[M + H]^+^	1.5	18.0	111.5	1.1	11.2	22.5
1519.868	Fengycin B1 C18 sFA	9.4	1518.861	[M + H]^+^	61.9	125.1	93.4	22.8	94.9	72.1
1477.819	Fengycin B2 C16 sFA	7.2	1476.813	[M + H]^+^	133.7	967	68.4	104.8	1091.3	15.8
Total fengycin					4953.3	27043.5	8669.2	5059.2	26,907	5827.5

+ represents isomers which showed two peaks; sFA - saturated fatty acid; usFA - unsaturated fatty acid

Extracted ion chromatograms (XIC) for m/z 1491.834 and m/z 1519.868 were assigned to fengycin B1 C16 and fengycin B1 C18 based on the MS/MS spectra (Table 3). Both XIC showed an additional peak that could not be assigned unambiguously by MS/MS. Most likely, the additional peaks correspond to fengycin isoforms with substitutions in the amino acid sequence and fatty acid chain length. Similarly, for XIC of surfactin C14 (m/z 1022.674), surfactin C15 (m/z 1036.69), and surfactin C16 (m/z 1050.706) additional signals were observed that could not assigned unambiguously by MS/MS spectra (Table 4).

**Table 4:** Comparison of peak area of surfactin LP from the dual culture of the three bacterial strains with Fos and their corresponding monoculture on a PDA plate

m/z	Name	RT (min)	Cal. MW	Positive ion	Peak area (x10^5^), dual culture			Peak area (x10^5^), monoculture		
					FZB42	GB03	BSn5	FZB42	GB03	BSn5
1008.658	Surfactin [Val2] C14	19.6	1007.651	[M + H]^+^	47.7	37.4	324.4	29.6	30	244.3
1022.674	Surfactin [Val2] C15	20.3	1021.667	[M + H]^+^	79.4	51	337	50.8	52.5	324.7
994.642	Surfactin [Val7] C13	18.45	993.635	[M + H]^+^	13.1	6.5	19.6	29.3	7.3	61.7
1008.656	Surfactin [Val7] C14	19.85	1007.652	[M + H]^+^	71.5	38.4	20.9	242.3	29	120.8
1022.675	Surfactin [Val7] C15	20.65	1021.668	[M + H]^+^	703.7	443.2	848.8	1576.3	400.2	887.6
1036.691	Surfactin [Val7] C16	21.2	1035.684	[M + H]^+^	577.3	135.5	1036	182	149.3	884.7
994.644	Surfactin C12	17.6	993.635	[M + H]^+^	139	89.1	47.7	75.8	86.5	30.9
1008.659	Surfactin C13	18.45	1007.652	[M + H]^+^	751.3	465.8	907.6	746.6	445.5	931.3
1022.674	Surfactin C14^+^	19.5	1021.668	[M + H]^+^	1205.8	618.5	1726	2936.9	710.8	2535.8
1022.674	Surfactin C14	19.75	1021.667	[M + H]^+^	3300.4	2203.7	14,749	2430.8	2079.1	958.8
1036.69	Surfactin C15^+^	20.45	1035.682	[M + H]^+^	6848.8	5067.8	6827.7	7677.9	4687	6277.7
1036.69	Surfactin C15	21.4	1035.683	[M + H]^+^	169	133.4	197.5	2090.4	114.7	456.3
1050.706	Surfactin C16^+^	21.45	1049.699	[M + H]^+^	221.5	64.1	251.8	557.3	69.1	476.6
1050.706	Surfactin C16	21.7	1049.699	[M + H]^+^	139.9	50.9	81.3	130.8	53.6	27.2
1064.722	Surfactin C17	22.35	1063.713	[M + H]^+^	37	20.9	36.5	129.2	15.6	43.3
Total surfactin					13779.4	9232.5	12940.1	18014.6	8737.5	13140.4

+ represents isomers which showed two peaks

### LC-MS/MS analysis of crude LP extracts from liquid culture

LC-MS/MS analysis of the cell free supernatants from liquid cultures revealed that the three bacterial strains FZB42, BSn5 and GB03 produced bacillomycin D, fengycin, and surfactin LP at varying levels (Fig. S3). Bacillomycin D was observed in the RT range of 1.8–8.2 min, while fengycins between 5.1 and 9.6 min and surfactins were detected at RT 17.4–22.8 min. Bacillomycin D was most abundant in FZB42 in liquid culture, followed by GB03, while it could be barely detected in BSn5 (Table S1). Several molecular ion peaks of bacillomycin D were detected, and the m/z 1031.545, 1045.555, and 1059.574 corresponding to the protonated molecular ion [M + H]^+^ were detected in high abundance in FZB42 and GB03, which had the strongest inhibitory activity against Fos (Fig. 2). In BSn5, it was, however, weakly detected. This was consistent with the plate culture result, suggesting a possible antifungal activity of bacillomycin D against Fos.

Additionally, a high abundance of fengycin and surfactin in the culture broth extracts of all strains was observed, with no significant difference in abundance observed across the strains. Both the saturated and unsaturated homologues of fengycin were observed in the broth culture extracts. Interestingly, unlike the LC-MS/MS analysis of the plate cultures, most of the fengycin variants were detected in the doubly charged ions [M + 2 H]^2+^ form in the liquid culture. This might be due to the higher abundance of fengycin in liquid than plate cultures. However, the overall abundance of bacillomycin D was lower than that of fengycin and surfactin in all strains. Among the three LP families, FZB42 and GB03 strains produced relatively more fengycin, while surfactin was the most abundant LP produced by the BSn5 strain. Fengycin B1 C16 m/z 746.4 [M + 2 H]^2+^ with saturated fatty acid was the most abundant fengycin isoform detected in FZB42 and GB03, while fengycin B2 C17 m/z 746.421 [M + 2 H]^2+^ with a saturated fatty acid variant was the most abundant isoform detected in BSn5 (Table S1).

## Discussion

This study aimed to investigate the inhibition of the mycoherbicide *Fusarium oxysporum* f.sp. *strigae* (Fos) by various bacterial species. *Bacillus* sp. strains FZB42, BSn5, and GB03 were found to significantly inhibit Fos growth in culture-based experiments. In this respect, we could show that LP released by *Bacillus* strains are a crucial factor in inhibiting Fos mycelial growth and spore germination, as well as causing structural distortion of Fos hyphae. The most notable effect of LP is the disruption of membrane integrity, leading to lysis of the mycelium and conidia of fungi, as well as perturbation of hyphal cells [21, 43]. This is due to the amphiphilic nature of LP, which enables them to interact with both hydrophobic and hydrophilic surfaces. LP also inhibit spore formation, induce bursts of reactive oxygen species, chromatin condensation, and organelle dysfunction [44, 45]. These findings strongly suggest that some plant-associated bacteria, including *Bacillus* sp., can interfere with Fos development through the release of LP. This may explain why Fos has shown inconsistent effectiveness in the biological control of Striga in the rhizosphere, where complex microbial interactions occur.

Apart from LP, bacteria can also produce other potent antifungal metabolites, such as polyketides (e.g., bacillaene, difficidin, macrolactin, bacilysin, bacteriocins) and siderophores (e.g., bacillibactin), which exhibit significant antimicrobial activity [15]. For example, FZB42 is well-known for producing a wide range of polyketides [46], making it a model strain for biocontrol and plant growth promotion. This strain devotes almost 10% of its genome to secondary metabolite synthesis and can produce massive amounts of LP [47, 48]. Similarly, GB03 and BSn5 also produce all three major LP families, with considerable amounts of potent antifungal properties. According to Cawoy et al. [49], bacterial strains that produce all three LP families, or at least the iturin families, are more efficient in inhibiting fungi. The simultaneous production of these antimicrobial metabolites enables bacteria to exhibit broad-range antagonism [50].

Our analysis of LP from both plate and liquid cultures suggests that bacillomycin D might be involved in the inhibition of Fos. This assumption is substantiated by the findings of [51], who confirmed that bacillomycin D is the crucial compound in *F. oxysporum* inhibition, while fengycin and surfactin showed no inhibitory effect. Although iturin and fengycin LP were reported to have strong antifungal activity, surfactin does not [20, 52]. Interestingly, fengycin was the most abundant LP in the liquid culture extracts of all three *Bacillus* strains analysed in this study, which is consistent with a report by Chen et al. [53].

Generally, LP are produced in large quantities under natural conditions, but external triggers such as stress, competition, and nutrition can significantly increase their production [49, 54]. Our results showed that the presence of Fos in dual culture triggered an increase in bacillomycin D production from the *Bacillus* strains, possibly through activation of signalling molecules as reported by Wakefield et al. [55]. Moreover, fungal metabolites produced by Fos in dual culture might also trigger LP production by bacteria. Additionally, fungal metabolites can lower the pH of the medium, and since the production of LP by bacteria is influenced by the pH of the medium [33, 56, 57]. This fact may also explain the increased LP production in the presence of Fos.

Bacillomycin D were induced in the plate culture experiment, when BSn5 was co-cultured with Fos, but in liquid culture they could be barely detected. Moreover, its abundance was lower in monoculture than in dual culture, indicating that bacillomycin D production might be induced by specific signalling events triggered by the presence of Fos. Conversely, bacillomycin D was present in higher abundance in both the dual and monoculture of GB03, suggesting that it could be produced in large quantities regardless of the presence of the fungus. Further experiments are needed to elucidate the influence of fungal metabolites on LP production by bacteria.

On the other hand, the result revealed that *P. phytofirmans* PsJN tended to have a positive effect on Fos development. *P. phytofirmans* PsJN is known for its potential plant growth promotion and disease resistance induction [29, 58], and its ability to produce various compounds such as phytohormones, siderophores and other secondary metabolites [59, 60]. In addition, the genome of *P. phytofirmans* PsJN contains a NRPS gene cluster responsible for the synthesis of LP [61]. Nonetheless, the key metabolites released by *P. phytofirmans* PsJN that might contribute to the speculated growth promotion effect on Fos remain elusive, necessitates further experimentation and validation.

## Conclusion

Our study provides evidence of the potent antagonistic effect of certain bacterial strains, including *Bacillus* sp. GB03, FZB42, and BSn5, on Fos development. These strains produce various isoforms of the three major LP families (iturin, fengycin, surfactin) and effectively inhibit Fos growth. Interestingly, bacillomycin D was induced in BSn5 and to a lower extent in FZB42, in response to the presence of Fos. Our findings emphasize the critical role of microbial interactions in shaping the efficacy of microbial assemblages for biological pest and disease control.

### Electronic supplementary material

Below is the link to the electronic supplementary material.

Supplementary Material 1

Supplementary Material 2

Supplementary Material 3

Supplementary Material 4

## Data Availability

The datasets generated and/or analysed during the current study are available from the corresponding author on reasonable request.
