# Supplementary figures and images for: Inhibitory activity of bacterial lipopeptides against Fusarium oxysporum f.sp. Strigae
Source: BMC Microbiol. 2024 Jun 27;24:227. doi: 10.1186/s12866-024-03386-2 (PMC11212183; doi:10.1186/s12866-024-03386-2)

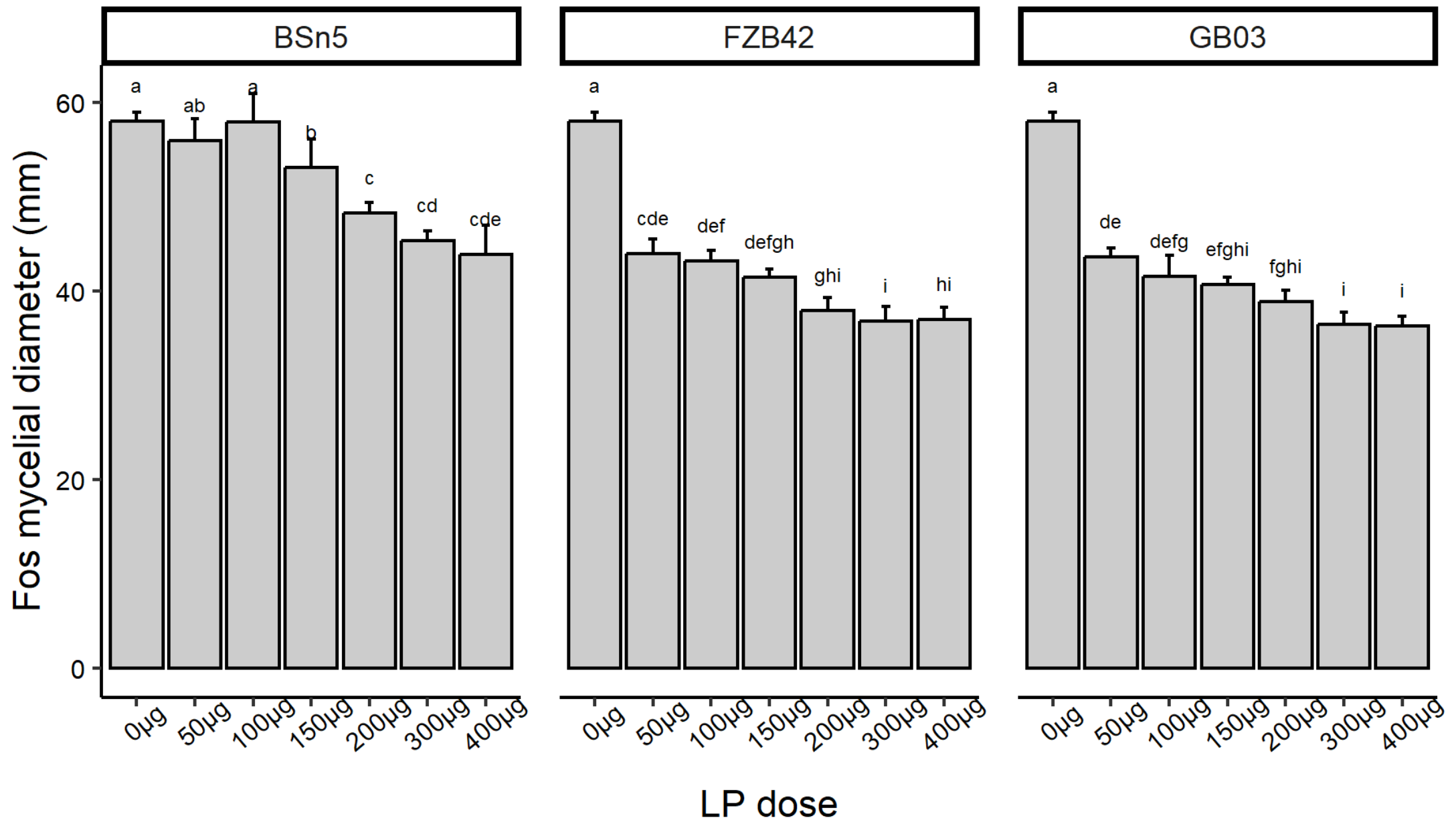

**Fig. S1.** Effect of various doses of crude LP from *Bacillus* sp. on Fos radial growth *in vitro*.

Supplement: Supplementary file 1 — Supplementary Material 1 [file 12866_2024_3386_MOESM1_ESM.pdf]
