# Supplementary material for: Inhibitory activity of bacterial lipopeptides against Fusarium oxysporum f.sp. Strigae
Source: BMC Microbiol. 2024 Jun 27;24:227. doi: 10.1186/s12866-024-03386-2 (PMC11212183; doi:10.1186/s12866-024-03386-2)
Supplement: Supplementary file 2 — Supplementary Material 2 [file 12866_2024_3386_MOESM2_ESM.pdf]

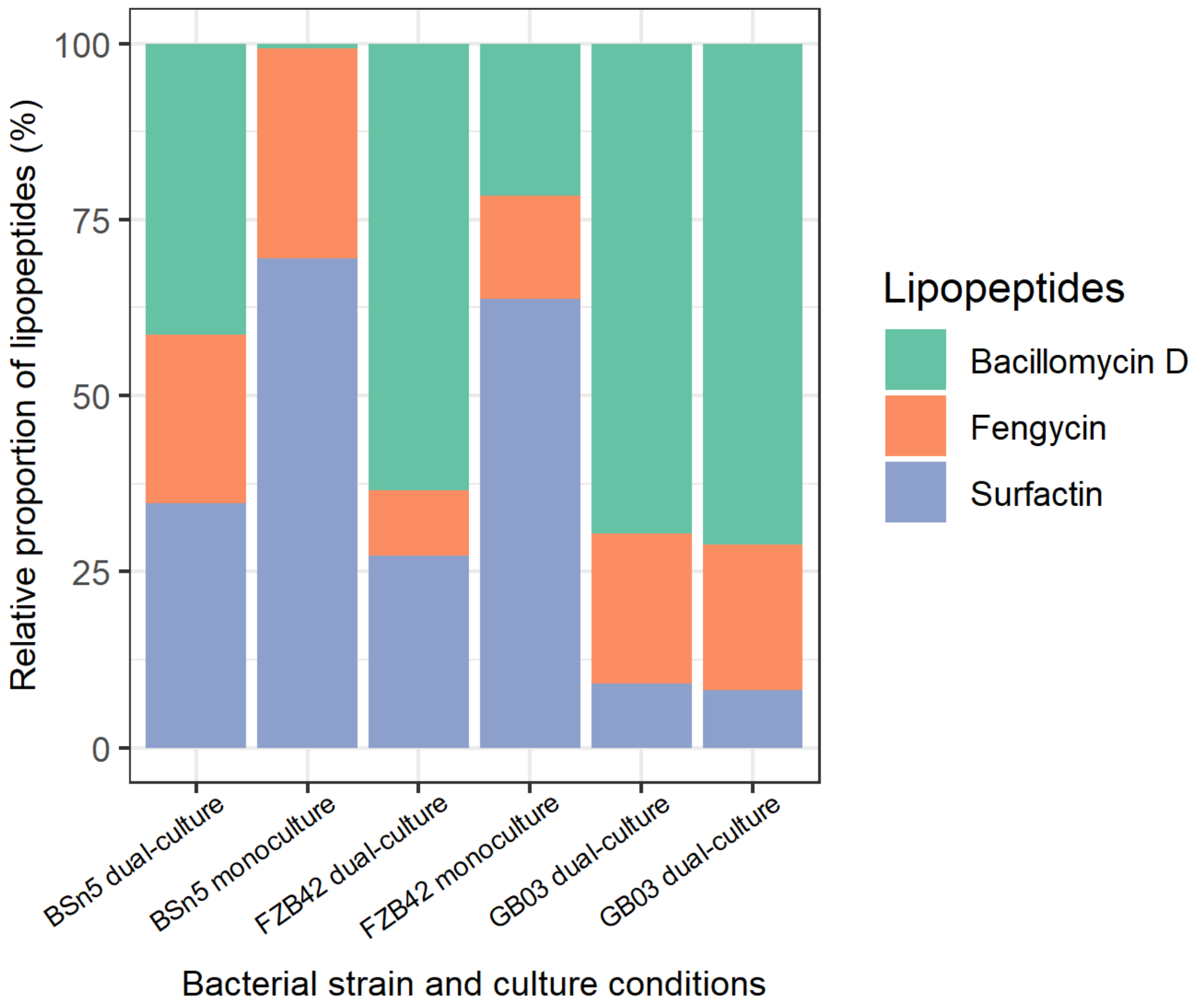

Fig. S2. Relative proportion of bacillomycin D, fengycin and surfactin LP abundance from the plate culture of *B. velezensis* GB03, *B. velezensis* FZB42 and *B. subtilis* BSn5 according to the peak area data retrieved from LC-MS analysis.
