# Supplementary material for: Inhibitory activity of bacterial lipopeptides against Fusarium oxysporum f.sp. Strigae
Source: BMC Microbiol. 2024 Jun 27;24:227. doi: 10.1186/s12866-024-03386-2 (PMC11212183; doi:10.1186/s12866-024-03386-2)
Supplement: Supplementary file 3 — Supplementary Material 3 [file 12866_2024_3386_MOESM3_ESM.pdf]

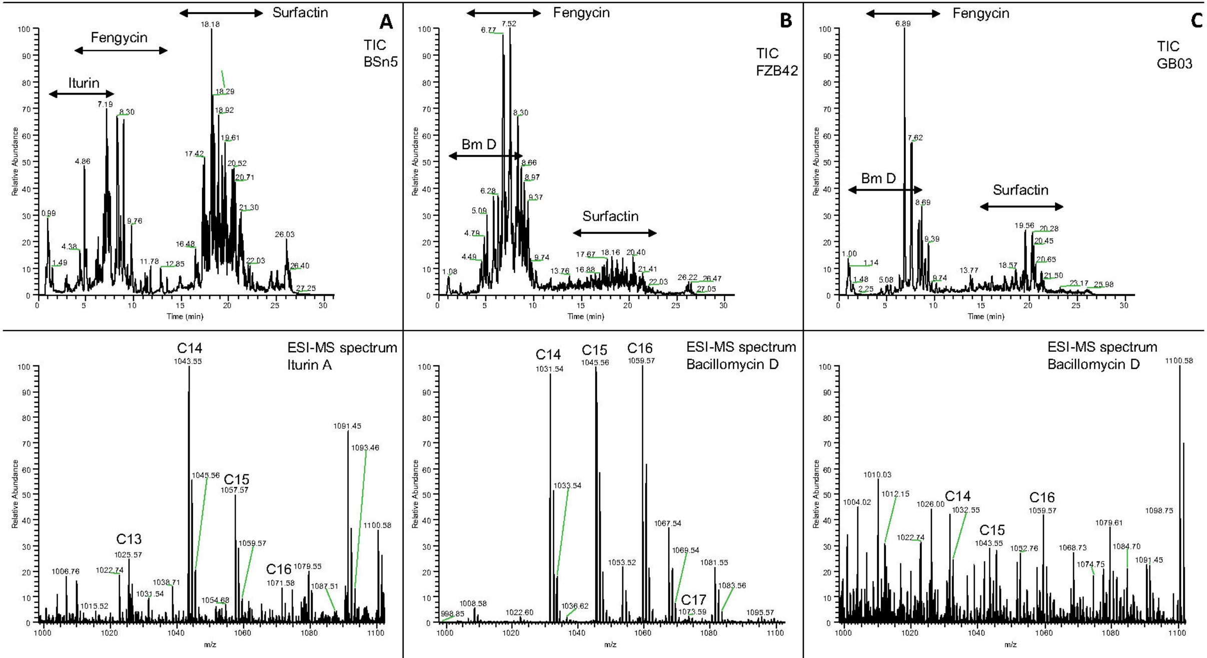

A
TIC
BSn5
Surfactin
Fengycin
Iturin
B
TIC
FZB42
Fengycin
Bm D
Surfactin
C
TIC
GB03
Fengycin
Bm D
Surfactin
Relative Abundance
Time (min)
ESI-MS spectrum
Iturin A
C13
C14
C15
C16
ESI-MS spectrum
Bacillomycin D
C14
C15
C16
C17
ESI-MS spectrum
Bacillomycin D
C14
C15
C16
m/z

Fig. S3. LC-ESI-MS analysis of the lipopeptide compounds produced by B. subtilis BSn5, *B. velezensis* FZB42 and *B. velezensis* GB03 in liquid culture. A: Total ion chromatogram (TIC, upper panel) and ESI-MS spectrum (lower panel) of the extracted Bacillomycin D/Iturin A lipopeptides from *B. subtilis* BSn5. The ESI-MS sum spectrum (m/z range 1000-1100) shows iturin A lipopeptides eluted in the time interval from 1 min – 8 min. Fatty acid chain length of different Iturin A lipopeptides is indicated. **B:** Total ion chromatogram (TIC, upper panel) and ESI-MS spectrum (lower panel) of the extracted Bacillomycin D lipopeptides *B. velezensis* FZB42. The ESI-MS sum spectrum (m/z range 1000-1100) shows Bacillomycin D lipopeptides eluted in the time interval from 1 min – 8 min. Fatty acid chain length of different Bacillomycin D lipopeptides is indicated. **C:** Total ion chromatogram (TIC, upper panel) and ESI-MS sum spectrum (lower panel) of the extracted Bacillomycin D lipopeptides *B. velezensis* GB03. The ESI-MS spectrum (m/z range 1000-1100) shows Bacillomycin D lipopeptides eluted in the time interval from 1 min – 8 min. Fatty acid chain length of different Bacillomycin D lipopeptides is indicated.
