# Supplementary material for: Inhibitory activity of bacterial lipopeptides against Fusarium oxysporum f.sp. Strigae
Source: BMC Microbiol. 2024 Jun 27;24:227. doi: 10.1186/s12866-024-03386-2 (PMC11212183; doi:10.1186/s12866-024-03386-2)
Supplement: Supplementary file 4 — Supplementary Material 4 [file 12866_2024_3386_MOESM4_ESM.docx]

**Table S1.** The peak area of bacillomycin D, fengycin and surfactin LP from the three *Bacillus* strains from methanolic extracts of the liquid culture.

| m/z | Name | RT (min) | Cal. MW | Positive ion | Peak area (x10^5^) | | |
| --- | --- | --- | --- | --- | --- | --- | --- |
|  |  |  |  |  | FZB42 | GB03 | BSn5 |
| 989.494 | Bacillomycin D C11 | 1.84 | 988.486 | [M+H]^+^ | 5.5 | 0.3 | 0.23 |
| 1003.51 | Bacillomycin D C12 | 2.61 | 1002.503 | [M+H]^+^ | 60.7 | 0.81 | 2.22 |
| 1017.525 | Bacillomycin D C13^+^ | 3.37 | 1016.517 | [M+H]^+^ | 33.5 | 0.5 | 0.61 |
| 1017.524 | Bacillomycin D C13 | 3.63 | 1016.517 | [M+H]^+^ | 36.3 | 0.09 | 0.27 |
| 1031.541 | Bacillomycin D C14 | 4.8 | 1030.534 | [M+H]^+^ | 16033.3 | 235.6 | 7.7 |
| 1045.555 | Bacillomycin D C15 | 5.73 | 1044.548 | [M+H]^+^ | 14689.3 | 129.4 | 1.7 |
| 1059.574 | Bacillomycin D C16 | 7.28 | 1058.566 | [M+H]^+^ | 13070.9 | 274.6 | 1.5 |
| 1073.588 | Bacillomycin D C17 | 8.19 | 1072.581 | [M+H]^+^ | 3837.1 | 22.7 | 1.7 |
| Total bacillomycin | |  |  |  | 47766.7a | 663.8ab | 15.9b |
| 718.389 | Fengycin A1 C14 sFA | 5.08 | 1434.763 | [M+2H]^2+^ | 5972.8 | 2794.4 | 1.2 |
| 1433.791 | Fengycin A1 C14 usFA | 7.42 | 1432.784 | [M+H]^+^ | 275.5 | 26.9 | 1.5 |
| 725.396 | Fengycin A1 C15 sFA | 5.77 | 1448.778 | [M+2H]^2+^ | 6407.3 | 1111.5 | 14.3 |
| 724.408 | Fengycin A1 C15 usFA | 8.36 | 1446.801 | [M+2H]^2+^ | 13260 | 6738.5 | 101.1 |
| 1463.804 | Fengycin A1 C16 sFA | 6.79 | 1462.797 | [M+H]^+^ | 38289.7 | 13508.5 | 92.1 |
| 1461.825 | Fengycin A1 C16 usFA | 9.19 | 1460.817 | [M+H]^+^ | 4755.9 | 422.7 | 490.9 |
| 739.413 | Fengycin A1 C17 sFA | 7.42 | 1476.812 | [M+2H]^2+^ | 21923.6 | 6409.7 | 274.7 |
| 746.421 | Fengycin A1 C18 sFA | 8.67 | 1490.828 | [M+2H]^2+^ | 20782.7 | 5802 | 38.7 |
| 717.400 | Fengycin A2 C15 usFA | 7.78 | 1432.785 | [M+2H]^2+^ | 2072.3 | 434.1 | 13.2 |
| 725.397 | Fengycin A2 C16 sFA | 6.32 | 1448.779 | [M+2H]^2+^ | 7288.8 | 4005.7 | 9.1 |
| 739.413 | Fengycin A2 C18 sFA | 8.12 | 1476.811 | [M+2H]^2+^ | 2097.2 | 1203.6 | 1.1 |
| 739.413 | Fengycin B1 C15 sFA | 6.51 | 1476.812 | [M+2H]^2+^ | 6538.5 | 1628.3 | 9.2 |
| 738.424 | Fengycin B1 C15 usFA | 8.96 | 1474.833 | [M+2H]^2+^ | 12547.9 | 7904.6 | 1037.8 |
| 746.421 | Fengycin B1 C16 sFA | 7.54 | 1490.828 | [M+2H]^2+^ | 52819.0 | 22700.2 | 1066.7 |
| 745.432 | Fengycin B1 C16 usFA^+^ | 9.63 | 1488.849 | [M+2H]^2+^ | 2828.7 | 1065 | 231.1 |
| 745.431 | Fengycin B1 C16 usFA | 8.89 | 1488.848 | [M+2H]^2+^ | 894 | 680.4 | 95.3 |
| 1505.85 | Fengycin B1 C17 sFA | 8.17 | 1504.843 | [M+H]^+^ | 6610.2 | 5057.8 | 1093.4 |
| 760.437 | Fengycin B1 C18 sFA | 9.39 | 1518.859 | [M+2H]^2+^ | 22427.2 | 7425.6 | 1395.4 |
| 731.415 | Fengycin B2 C15 usFA | 8.40 | 1460.815 | [M+2H]^2+^ | 999.4 | 2273.3 | 808.8 |
| 739.413 | Fengycin B2 C16 sFA | 7.11 | 1476.812 | [M+2H]^2+^ | 19504.6 | 9747.2 | 207.5 |
| 746.421 | Fengycin B2 C17 sFA | 8.04 | 1490.828 | [M+2H]^2+^ | 16136.4 | 876.6 | 1592.8 |
| Total fengycin | | | | | 264431.6 | 101816.5 | 8576.2 |
| 1008.66 | Surfactin C13^+^ | 18.5 | 1007.653 | [M+H]^+^ | 2985.4 | 69.6 | 2432.5 |
| 1008.659 | Surfactin [Val2, Val7] C15 | 19.80 | 1007.652 | [M+H]^+^ | 2249.2 | 416.4 | 4156.5 |
| 1022.675 | Surfactin [Val2] C15 | 19.92 | 1021.668 | [M+H]^+^ | 1463 | 334 | 4907.1 |
| 994.644 | Surfactin [Val7] C13 | 18.40 | 993.637 | [M+H]^+^ | 283.3 | 67.1 | 1470 |
| 1022.676 | Surfactin [Val7] C15 | 20.49 | 1021.668 | [M+H]^+^ | 3480.3 | 2253.1 | 2781.6 |
| 1036.691 | Surfactin [Val7] C16 | 21.71 | 1035.684 | [M+H]^+^ | 682.9 | 20.9 | 1835.8 |
| 1050.706 | Surfactin [Val7] C17 | 22.79 | 1049.699 | [M+H]^+^ | 664 | 37.5 | 995.8 |
| 994.643 | Surfactin C12 | 17.42 | 993.636 | [M+H]^+^ | 4073.4 | 629.5 | 5.5 |
| 1008.66 | Surfactin C13 | 18.25 | 1007.652 | [M+H]^+^ | 5226.7 | 1341.4 | 927.4 |
| 1022.675 | Surfactin C14 | 19.65 | 1021.667 | [M+H]^+^ | 11948.1 | 6709.5 | 3766.3 |
| 1022.675 | Surfactin C14 | 17.74 | 1021.668 | [M+H]^+^ | 44.3 | 7.2 | 18448 |
| 1036.69 | Surfactin C15 | 20.40 | 1035.683 | [M+H]^+^ | 13728.7 | 3202.2 | 427.7 |
| 1036.69 | Surfactin C15 | 18.98 | 1035.683 | [M+H]^+^ | 33.1 | 1.4 | 4093.6 |
| 1050.706 | Surfactin C16 | 21.31 | 1049.698 | [M+H]^+^ | 13620 | 3550.8 | 1291.8 |
| 1050.707 | Surfactin C16 | 19.70 | 1049.7 | [M+H]^+^ | 21.4 | 3.1 | 1840 |
| 1064.722 | Surfactin C17 | 22.12 | 1063.714 | [M+H]^+^ | 2477 | 155 | 2242.5 |
| Total surfactin | | | | | 62980.7 | 18798.7 | 51621.9 |

+ represents isomers which showed two peaks; sFA - saturated fatty acid; usFA - unsaturated fatty acid. Means followed by the same letter are not significantly different (Kruskal-Wallis test with Dunn’s multiple comparison test, P<0.05).
